# Enhancing the Photovoltaic Properties via Incorporation of Selenophene Units in Organic Chromophores with A_2_-π_2_-A_1_-π_1_-A_2_ Configuration: A DFT-Based Exploration

**DOI:** 10.3390/polym15061508

**Published:** 2023-03-17

**Authors:** Muhammad Nadeem Arshad, Iqra Shafiq, Muhammad Khalid, Mohammad Asad, Abdullah M. Asiri, Maha M. Alotaibi, Ataualpa A. C. Braga, Anish Khan, Khalid A. Alamry

**Affiliations:** 1 Center of Excellence for Advanced Material Research (CEAMR), King Abdulaziz University, P.O. Box 80203, Jeddah 21589, Saudi Arabia; 2 Chemistry Department, Faculty of Science, King Abdulaziz University, P.O. Box 80203, Jeddah 21589, Saudi Arabia; 3 Institute of Chemistry, Khwaja Fareed University of Engineering & Information Technology, Rahim Yar Khan 64200, Pakistan; 4 Centre for Theoretical and Computational Research, Khwaja Fareed University of Engineering & Information Technology, Rahim Yar Khan 64200, Pakistan; 5 Departamento de Química Fundamental, Instituto de Química, Universidade de Sao Paulo, Av. Prof. Lineu Prestes, 748, Sao Paulo 05508-000, Brazil

**Keywords:** polymer organic solar cells, selenophene units, photovoltaic properties, A_2_-π_2_-A_1_-π_1_- A_2_ configuration, DFT, open-circuit voltage

## Abstract

Currently, polymer organic solar cells (POSCs) are widely utilized due to their significant application, such as low-cost power conversion efficiencies (PCEs). Therefore, we designed a series of photovoltaic materials (**D1, D2, D3, D5** and **D7**) by the incorporation of selenophene units (*n* = 1–7) as π_1_-spacers by considering the importance of POSCs. Density functional theory (DFT) calculations were accomplished at MPW1PW91/6-311G (d, p) functional to explore the impact of additional selenophene units on the photovoltaic behavior of the above-mentioned compounds. A comparative analysis was conducted for designed compounds and reference compounds (**D1**). Reduction in energy gaps (∆E = 2.399 − 2.064 eV) with broader absorption wavelength (λ_max_ = 655.480 − 728.376 nm) in chloroform along with larger charge transference rate was studied with the addition of selenophene units as compared to **D1**. A significantly higher exciton dissociation rate was studied as lower values of binding energy (E_b_ = 0.508 − 0.362 eV) were noted in derivatives than in the reference (E_b_ = 0.526 eV). Moreover, transition density matrix (TDM) and density of state (DOS) data also supported the efficient charge transition origination from HOMOs to LUMOs. Open circuit voltage (*V_oc_*) was also calculated for all the aforesaid compounds to check the efficiency, and significant results were seen (1.633–1.549 V). All the analyses supported our compounds as efficient POSCs materials with significant efficacy. These compounds might encourage the experimental researchers to synthesize them due to proficient photovoltaic materials.

## 1. Introduction

Usually, inorganic solar cell structures were prepared by gallium arsenide (GaAs) and silicon (Si). They have attained great consideration because of their stability and high energy conversion efficiencies moving towards their theoretical limits for their precise bandgaps. Silicon-based solar cells (Si-SCs) used since today have a high proficiency of about 46% [1,2]. However, with the passage of time, the utilization of silicon has been reduced to a remarkable extent due to its fixed composition, non-tunable energy levels, brittleness, high cost and a limited number of atoms and compact structure. Consequently, researchers are now trying to substitute the silicon-based materials. A number of advantages, such as an easy processability, low weight, cheaper and adaptable energy levels, have been observed for organic solar cells [3,4,5]. Organic SCs are fabricated on the elementary structure of inorganic SCs by substituting n-type and p-type with donors and acceptors [6,7,8]. Dye-sensitized solar cells (DSSCs) were gaining considerable attention due to their significant photovoltaic performances. Moreover, DSSCs have low costs, great stability and tunable visual properties, such as transparency and color [9,10]. The dye in DSSCs catches special attention due to its ability to convert light into electricity by photoexcitation phenomena [11]. Further, DSSCs are reported with significant efficiency, facile synthetic route and purification. DSSCs have many tunable optical properties through easy modifications in chemical structures [12]. Moreover, the photovoltaic world is enriched with many interesting materials, such as organic solar cells’ fullerene derivatives such as PC61BM, PC71BM and ICBA. The fullerene-based OSCs are found with the following remarkable properties: tunable energy levels, simple processability, lightweight, large area of fabrication and mechanical flexibility [3,4,5,13,14,15]. Owing to the following limitations: limited absorption behavior and larger molecular weight of fullerene [16] the main focus of researchers has been moved towards non-fullerene organic solar cells (NF-OSCs) as they obtained many significant findings in NF-OSCs [17,18,19]. Recently, NF-OSCs gained considerable attention from researchers due to their high flexibility, pellucid nature and tunable energy level [14,20,21]. The literature revealed many reports where organic systems with the following various architectures: donor-acceptor, donor-π-linker-acceptor-π-spacer-donor, donor-π spacer-acceptor, donor-donor-π-acceptor, acceptor-π-spacer-donor-π-linker-acceptor and donor-acceptor-π-acceptor. These architectures are widely utilized for significant OSCs materials [22,23]. Further, NFAs are blended with donor polymers and, subsequently, optical absorption properties are improved. These phenomena aided to enhance the PCE of single-junction cells by up to 17% [24]. Apart from this, there is also found some of the following features of non-fullerene acceptors (NFAs) derivatives: facile synthesis [13,21], material design [25,26] and realization of the mechanism and their optimization [27,28]. They have contained the approach for rationalizing significant PCEs over 18% [29]. In addition, stability tests of NFAs have been performed and data reveal that NFAs-based OSCs may obtain a life span of up to 10 years, which reveals their promising values for practical application [30,31]. The NF-OSCs are categorized into the following two groups: small molecular acceptor (SMA) and polymer organic solar cells (POSCs) [6,7,32]. Recently, the POSCs gained the attention of the researchers as efficient photovoltaic materials due to their improved open-circuit voltage (*V_oc_*) and significantly low price PCEs [33,34,35,36]. In the current century, devotion is paid to engineering polymer bulk hetero-junction (BHJ) SCs initiated on fullerene donors and fullerene-free polymeric acceptors, progressing efficiency to 8.3% [37]. Conjugative POSCs attracted significant attention due to their efficient ability to absorb sunlight and tunable electronic characteristics and good PCEs [38,39]. The π-resonance and behavior of substituents control the band gap, influence the charge transfer and enhanced photovoltaic properties [22,40]. Different strategies and criteria are present in the literature, which imply fine-tuning of acceptor-donor blend to boost UV-Visible absorption, resulting in uplifted short circuit current density (*J_sc_*), lessening of the energy gap between HOMO and LUMO, optimized morphology of blend to increase the fill factor (FF) and open-circuit voltage (*V_oc_*).

Keeping in consideration the aforementioned aspects, here we have taken an NF-based synthesized parent compound, namely, 2,2’-((2E,2’E)-((6,6’-(2,5-difluoro-1,4-phenylene)bis(4,4-bis(2-ethylhexyl)-4H-cyclopenta [1,2-b:5,4-b’]dithiophene-6,2-diyl))bis(methanylylidene))bis(3-oxo-2,3-dihydro-1H-indene-2,1-diylidene))dimalononitrile abbreviated as **DF-PCIC** [41] having A_2_-*π*_2_-A_1_-*π*_1_-A_2_ configuration. After replacing 4,4-dimethyl-4H-cyclopenta [1,2-b:5,4-b’]dithiophene with a selenophene ring as the first π-spacer (*π*_1_) and via substituting both the terminal acceptors with benzothiophene acceptors (A_2_) on both sides, the parent compound is modified into a reference compound, which is denoted as **D1**. From the literature, we found that selenophene units and benzothiophene acceptors can lower the LUMO level with an unchanged HOMO level and effectively improve the *V_oc_* and *J_sc_* in POSCs [42,43,44,45]. Therefore, in the current study, we performed molecular engineering of **D1** with a selenophene ring and benzothiophene acceptors to improve the *V_oc._* **D2**, **D3**, **D5** and **D7** compounds. These compounds were designed by the incorporation of selenophene units (n = 2–7) in this reference (**D1)**. The influence of structural modifications on electronic and optical behavior is explored in this research paper through DFT. It is predicted that the designed derivatives might be beneficial for the engineering of highly efficient OSCs.

## 2. Computational Procedure

The Gaussian 09 program [46] was employed to perform the calculations of current research work. First of all, geometries of aforesaid chromophores were optimized at MPW1PW91 functional with 6–311 G (d, p) basis set to obtain geometries at true minima. With the aid of Gauss View 5.0 [47], the input files were drawn. To find the photovoltaic properties of selenophene derivatives (**D1, D2, D3, D5** and **D7**), various kinds of analyses such as DOS, *V_oc_,* E_b_, *µ*_tot_ and GRPs were accomplished at the aforesaid level of DFT by utilizing the optimized structures. Nevertheless, the following key electronic properties: TDM, FMO analysis and optical properties, were investigated through TD-TDF at the above-mentioned functional. To interpret the results from output files, multiple software such as; Avogadro [48], Gauss Sum [49], Chemcraft [50], Multiwfn 3.7 [51] and PyMOlyze 2.0 [52], Origin 8.5 program [53] were utilized.

## 3. Results and Discussion

**Scheme 1:**
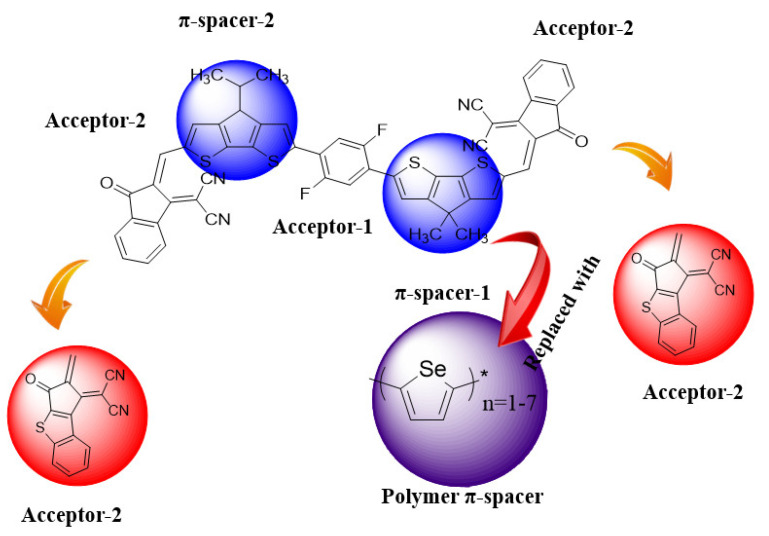
The sketch map of designed chromophores (**D1, D2, D3, D5** and **D7**). From parent molecule (**DF-PCIC**).

**Figure 1:**
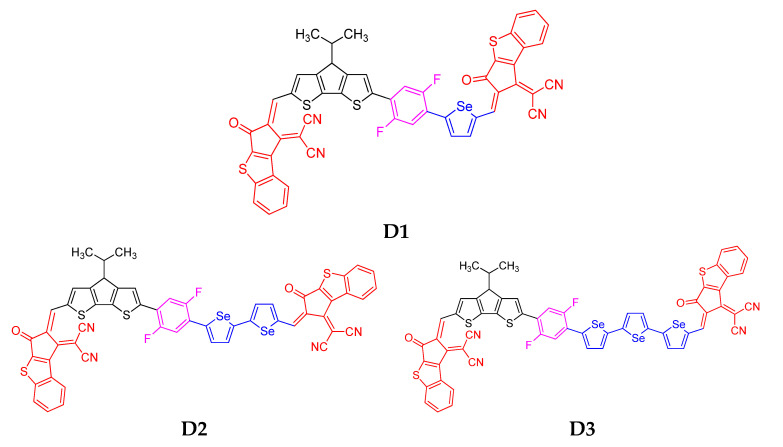

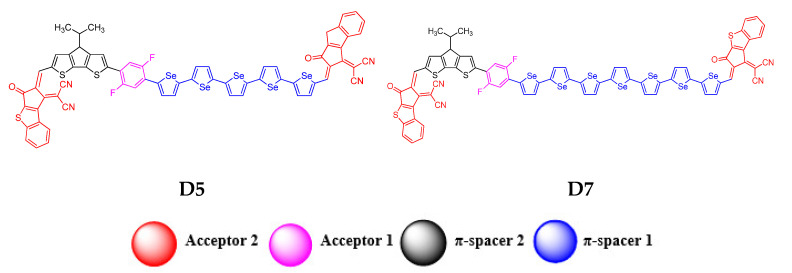
The 2D geometries of **D1**, **D2**, **D3**, **D5** and **D7**.

**Figure 2:**
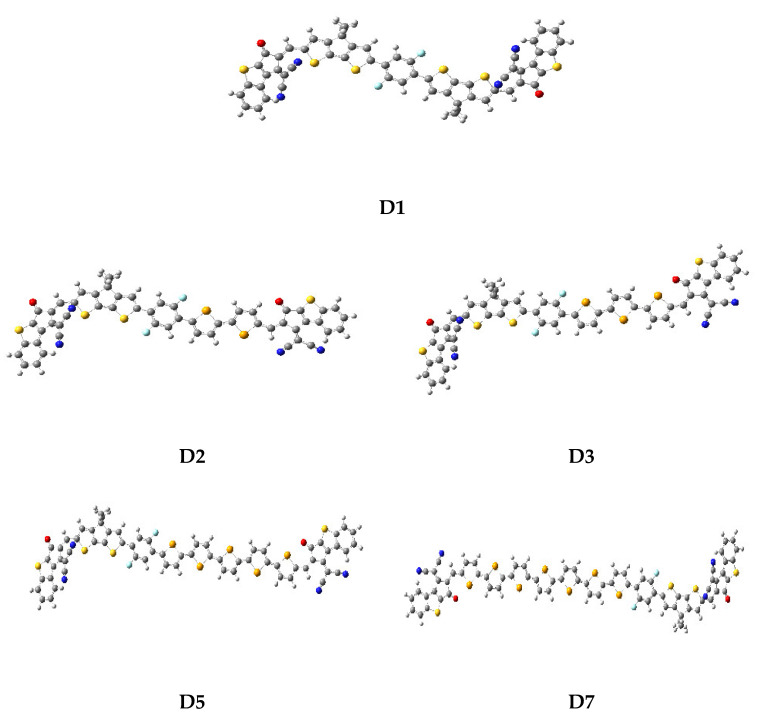
True minima structures of selenophene-based derivatives.

Nowadays, polymer organic solar cells (POSCs) are widely utilized as photovoltaic devices due to their low-cost sunlight conversion efficiencies [54,55]. The literature is flooded with many reports in which small units, such as thiophene, selenophene and imidazole, etc. were utilized to improve the charge transference properties of organic systems [8,42,56]. From the literature, we found that by replacing the sulfur with a selenium atom, a significant reduction in band gap can be achieved [57] The current approach aims to explore the effect of selenophene unit on charge transference rate between orbitals and also described the influence on the photovoltaic properties of organic systems. For this purpose, a synthesized fullerene-free organic system (**DF-PCIC**) is chosen as a parent molecule to design reference compounds (**D1**) with an A_2_-*π*_2_-A_1_-*π*_1_-A_2_ framework. First of all, the *π*-bridge (4H-cyclopenta [1,2-b:5,4-b’]dithiophene) on the one side of **DF-PCIC** is replaced with selenophene and kept the other side preserved. The terminal acceptors (2-(2-methylene-3-oxo-2,3-dihydro-1H-inden-1-ylidene)malononitrile) in **DF-PCIC** are replaced with a benzothiophene-based acceptor (2-(2-methylene-3-oxo-2,3-dihydro-1H-benzo[b]cyclopenta[d]thiophen-1-ylidene)malononitrile) in order to improve the electron-withdrawing effect in **D1** (Scheme 1 and Figure 1). Then **D1** is considered a reference molecule, and further, it is utilized to design other derivatives (**D2, D3, D5** and **D7**). The **D2** and **D3** are designed by introducing two and three units of selenophene, respectively, keeping the same acceptors as shown in Figure 1. Furthermore, to explore the effect of the high number of selenophene units on the photovoltaic responses of POSCs, we introduce five and seven units of selenophene, respectively, in **D5** and **D7** (Figure 1). The IUPAC names of reference (**D1**) and their derivatives (**D2, D3, D5** and **D7**) are explained in the Supplementary File. The true minima structures of the above-mentioned POSCs are displayed in Figure 2, and their cartesian coordinates are tabulated in Tables S1–S5.

## 3.1. Frontier Molecular Orbital (FMO) Analysis

FMO analysis is deliberated as a leading method to estimate the electronic properties of organic systems [58,59]. FMOs are indispensable to accelerating the transmission of electric current and provide the properties of photovoltaic cells with the aptitude for conducting electronic charges [60,61]. In accordance with the valance band theory, HOMO is considered a valence band, while LUMO is regarded as a conduction band. The band gap of HOMO and LUMO is a substantial factor in elucidating several quantum chemical parameters such as chemical reactivity, charge transfer, UV-Visible spectrum, chemical stability and electronic properties [62,63,64]. The potent photovoltaic response is indicated by less energy difference that reasonably determines the efficiency of a compound [65]. The calculated HOMO-LUMO energies as well as their energy gaps for designed molecules, are composed in Table 1.

Table 1 illustrates that the energies of HOMO/LUMO for **D1** are established to be −5.845/−3.380 eV with a 2.465 eV energy gap. While the HOMO/LUMO energies for its derivatives (**D2, D3, D5** and **D7**) are recorded as −5.744/ −3.345, −5.636/ −3.329, −5.472/ −3.305 and −5.361/ −3.297 eV and their energy gaps are computed as 2.399, 2.307, 2.167 and 2.064 eV, respectively. The declining *E_gap_* noticed from **D1** to **D7** might be due to the continual addition of selenophene monomer in *π*_1_ in each designed compound extending the conjugation and boosting the charge transfer. The charge transfer of a chromophore is indirectly related to the *E_gap_*, i.e., the lower the *E_gap_*, the greater the charge transfer and vice versa [66,67]. The overall declining trend of *E_gap_* is viewed as **D1** (2.465 eV) > **D2** (2.399 eV) > **D3** (2.307 eV) > **D5** (2.167 eV) > **D7** (2.064 eV). The effective charge mobility from acceptor−2 to acceptor−1 through *π*-spacer along with the lowest *E_gap_* among the molecular orbitals are noticed in **D7** chromophore than other designed molecules, which emerged to be an efficient material for use in photovoltaic devices.

**Figure 3:**
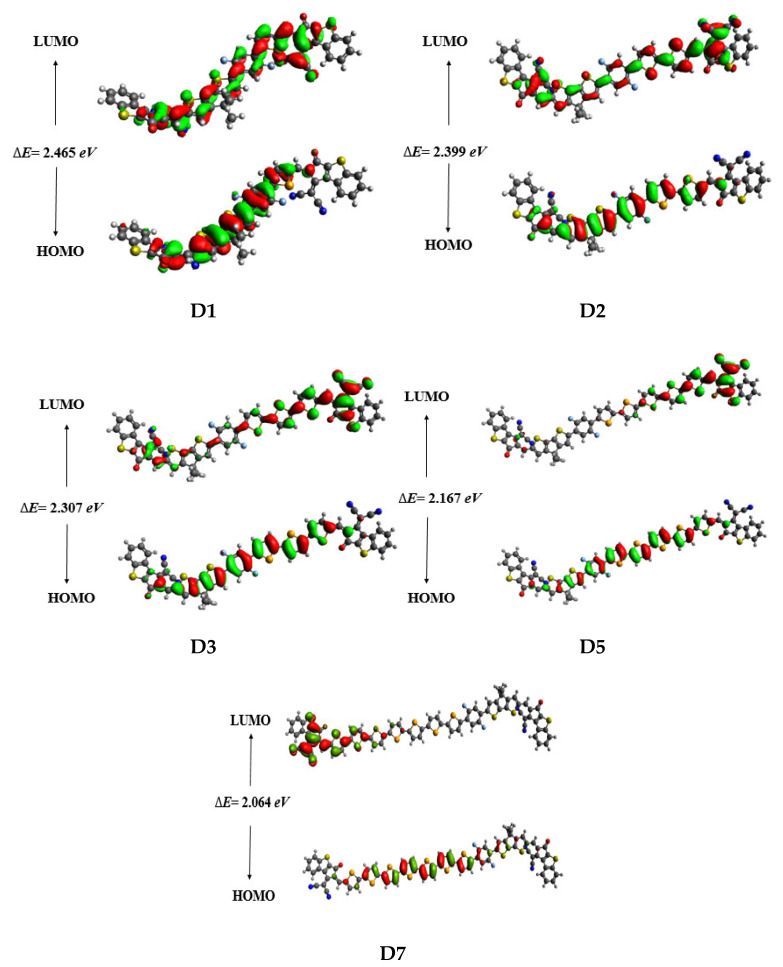
FMO diagrams of studied compounds.

The FMOs contour surface diagrams are illustrated in Figure 3, which expresses the distribution of electronic clouds over the molecules. In **D1** and **D2**, charge density is significantly concentrated on the central part, while a little bit of electronic density is noticed on terminal acceptor entities in HOMO and LUMO. In **D3, D5** and **D7**, HOMO is majorly concentrated on the acceptor−2 and *π*-bridge, whereas LUMO is mainly located in electron-deficient end-capped groups. Hence, the analyzed molecular systems showed charge transmission from acceptor−2 to acceptor−1 through the *π*-bridge. The energies of HOMO-1/LUMO+1 and HOMO-2/LUMO+2 are illustrated in Table S6, while their FMO diagrams are displayed in Figures S1–S5. Almost the same phenomena for energies and charger transference are seen between higher orbitals (HOMO-1/LUMO+1 and HOMO-2/LUMO+2).

## 3.2. Optical Properties

**Figure 4:**
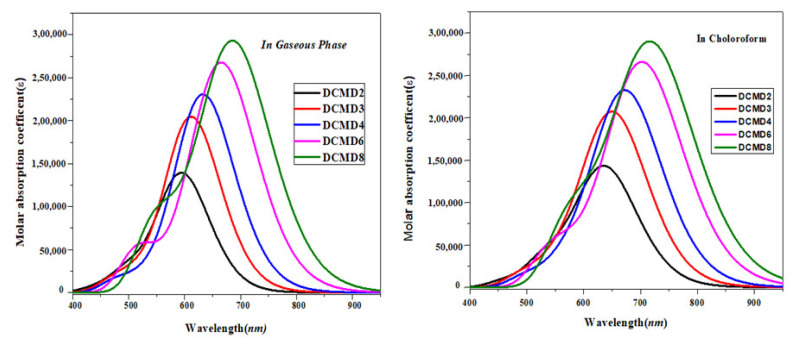
Absorption spectra of entitled compounds i.e.

A UV-Visible analysis is a significant tool to elucidate the probability of charge transference, the nature of electronic transitions and contributing configuration in transitions within the chromophores [68,69]. TD-DFT calculations were performed at MPW1PW91/ 6–311 G (d,p) level in chloroform and gaseous phase to assess the photophysical properties of the designed chromophores. The main outcomes of oscillator strength (*f_os_*), transition energy (*E*) and maximum absorption wavelengths (*λ_max_*) are collected in Table 2 and Table 3 in gas and chloroform, respectively, and their graphs are presented in Figure 4. Moreover, other results are exhibited in Tables S8–S17.

Table 2 shows that the absorption values of **D1**, **D2, D3, D5** and **D7** in the range of 596.393–691.953 nm with 1.872–3.708 oscillation strength and 1.791–2.078 eV transition energy in the gas phase are found. The strongest absorption of 691.953 nm is exhibited by **D7,** which might be because of the extended conjugation and effective intramolecular charge transfer. The increasing *λ_max_* order is found to be **D1** (596.393 nm) < **D2** (613.722 nm) < **D3** (635.490 nm) < **D5** (668.702 nm) < **D7** (691.953 nm).

In chloroform solvent, the *λ_max_* values of titled molecules are obtained to be 639.390, 655.480, 676.474, 708.521 and 728.376 nm for **D1**, **D2, D3, D5** and **D7**, respectively (Table 3). The values of *λ_max_* are observed as **D7** (728.376 nm) > **D5** (708.521 nm) > **D3** (676.474 nm) > **D2** (655.480 nm) > **D1** (639.390 nm). This enhancement might be regarded as the continuous addition of selenophene units in the first *π*-spacer (*π*_1_) in each derivative, which results in extending the conjugation and boosting the charge transfer. Meanwhile, the absorption wavelength of these molecules is compared in the solvent phase to that in the gaseous phase, and it is noticed that *λ_max_* in chloroform is also examined to be greater than the gas phase due to the solvent effect. Overall, the maximum bathochromic shift is observed for **D7** in both phases, so these designed chromophores can be regarded as excellent solar cell material for future use.

### 3.3. Global Reactivity Parameters (GRPs) Investigations

To investigate the stability and reactivity of a molecule, GRPs are calculated through the energies of HOMOs and LUMOs. The global softness (*σ*), electron affinity (*EA*), global hardness (*η*), global electrophilicity index (*ω*), chemical potential (*μ*), ionization potential (*IP*) and electronegativity (*X*) were computed by using the band gap of HOMO and LUMO [70,71,72,73,74]. The following Equations (1) and (2) are used to calculate *EA* and *IP*.

*IP* = −*E_HOMO_* (1)

*EA* = −*E_LUMO_* (2)

Koopmans’s theorem is utilized to determine *σ*, *ω*, *η*, *μ* and *X* [75].

(3) $$X=\frac{\left[IP+EA\right]}{2}$$

(4) $$\eta =\frac{\left[IP-EA\right]}{2}$$

(5) $$\mu =\frac{E_{\mathrm{HOMO}}+E_{\mathrm{LUMO}}}{2}$$

(6) $$\sigma =\frac{1}{2\eta }$$

(7) $$\omega =\frac{\mu^{2}}{2\eta }$$

The above parameters were obtained utilizing Equations (1)–(7), and these results are displayed in Table 4. The chemical potential of a molecule expresses the stability and reactivity of a specie. *IP* signifies the electron donating and accepting ability, which is the energy requires to eradicate the electron from HOMO. The energy gap, chemical potential, stability and hardness are inversely associated with reactivity while directly to one another [69]. Moreover, the stability of a molecule depends upon the electronegativity and the position of its substituents with respect to the electronegative atom [76]. Thus, the molecule with greater energy difference is considered harder, which shows low reactivity and high kinetic stability.

The *IP* values are noted to be greater in magnitude than *EA* values. The hardness values are noticed as 1.233, 1.199, 1.154, 1.084 and 1.167 eV for **D1**, **D2, D3, D5** and **D7**, respectively, and its descending order is found in studied molecules as **D1** > **D2** > **D7** > **D3** > **D5**. The hardness of a molecule is directly linked with the *E_gap_* and inversely related to the reactivity. Therefore, a chromophore with a greater energy gap is considered harder and more stable [77]. Another factor that discloses the reactivity of molecules is softness, which is directly associated with polarizability [78]. The softness value calculated for **D5** is observed to be 0.461 eV, which reduces to 0.434 eV in **D3** and further declines to 0.428 eV for **D7**, while the least value (0.417 eV) is noted in **D2**. Interestingly, the highest value of softness (0.461 eV) is viewed in **D5**, which might be due to an increase in conjugation due to an extended *π*-spacer. Thus, it is regarded as the most polarizable and exhibits good photovoltaic properties for all the said chromophores.

### 3.4. The Density of State (DOS) Analysis

**Figure 5:**
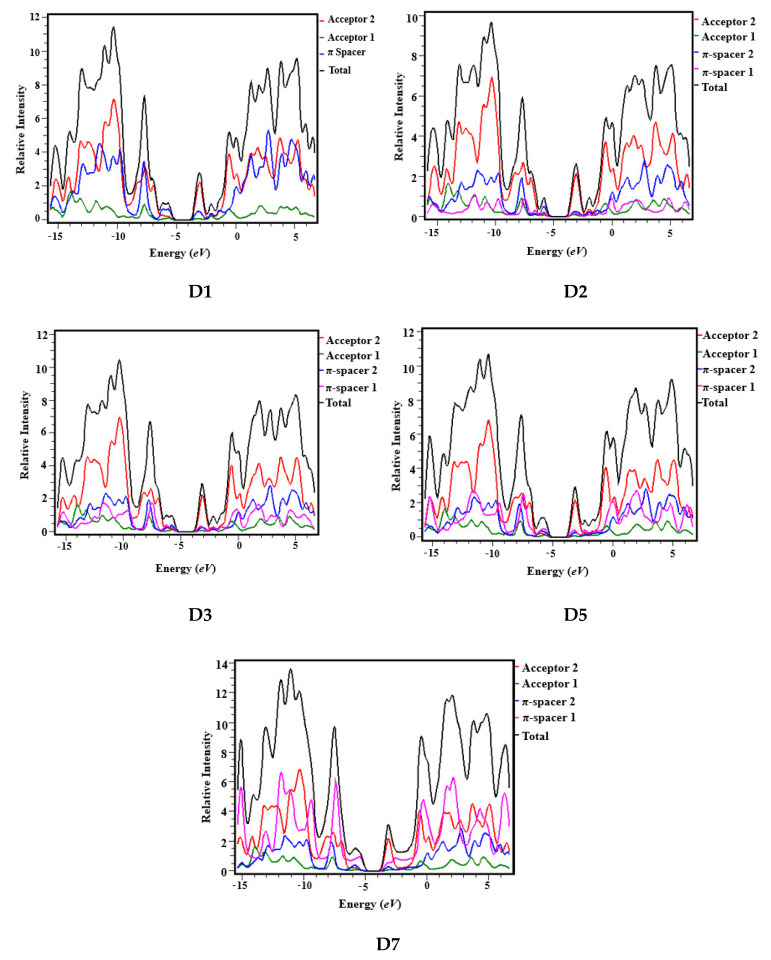
Graphical representation of DOS spectra for **D1**, **D2, D3, D5** and **D7**.

The DOS analysis is accomplished to estimate the contribution of each fragment of the molecule in the total electronic distribution and absorption band [42,79]. To perform this analysis, the designed molecules are partitioned into four fragments, i.e., acceptor-2, *π*-spacer-2, acceptor-1 and *π*-spacer-1. DOS was carried out for **D1**, **D2, D3, D5** and **D7** to support the insights obtained from FMO exploration [80]. The DOS pictographs are displayed in Figure 5, where each fragment is presented in different colors (acceptor-2 with red, acceptor-1 with green, *π*-spacer-2 with blue and *π*-spacer-1 with pink lines). The pattern of electronic charge dissemination is altered by changing acceptor moieties and extending the *π*-spacer, which is justified by the DOS percentage of HOMO-LUMO. Herein, acceptor-1 depicted 14.1, 14.7, 13.0, 7.2 and 4.2% charge distribution pattern to HOMO, whereas 9.8, 7.1, 3.8, 1.0 and 0.2% to LUMO for **D1**, **D2, D3, D5** and **D7**, respectively. Likewise, acceptor-2 showed charge contributions as follows: 22.4, 18.5, 14.1, 7.4 and 3.7% to HOMO, while 60.0, 53.4, 59.1, 61.8 and 64.0% to LUMO. Furthermore, *π*-spacer-1 demonstrates an electronic distribution pattern as follows: 8.7, 24.6, 45.3, 74.8 and 87.3% to HOMO, whereas 15.3, 26.9, 30.0, 35.1 and 35.5% to LUMO in **D1**, **D2, D3, D5** and **D7**, correspondingly. Similarly, *π*-bridge-2 manifested a pattern of electronic distribution as follows: 54.8, 42.2, 27.6, 10.6 and 4.8% to HOMO, while 60.0, 53.4, 59.1, 61.8 and 64.0% to LUMO, accordingly. It is clear from these outcomes that HOMO is predominantly located on acceptor-1, while LUMO mainly resides on acceptor-2 in the aforementioned compounds. Overall, the pattern of electronic charge distribution unveils that a significant amount of charge is shifted from HOMO to LUMO, exhibiting them as promising candidates for fullerene-free OSCs.

### 3.5. Transition Density Matrix (TDM) Study

**Figure 6:**
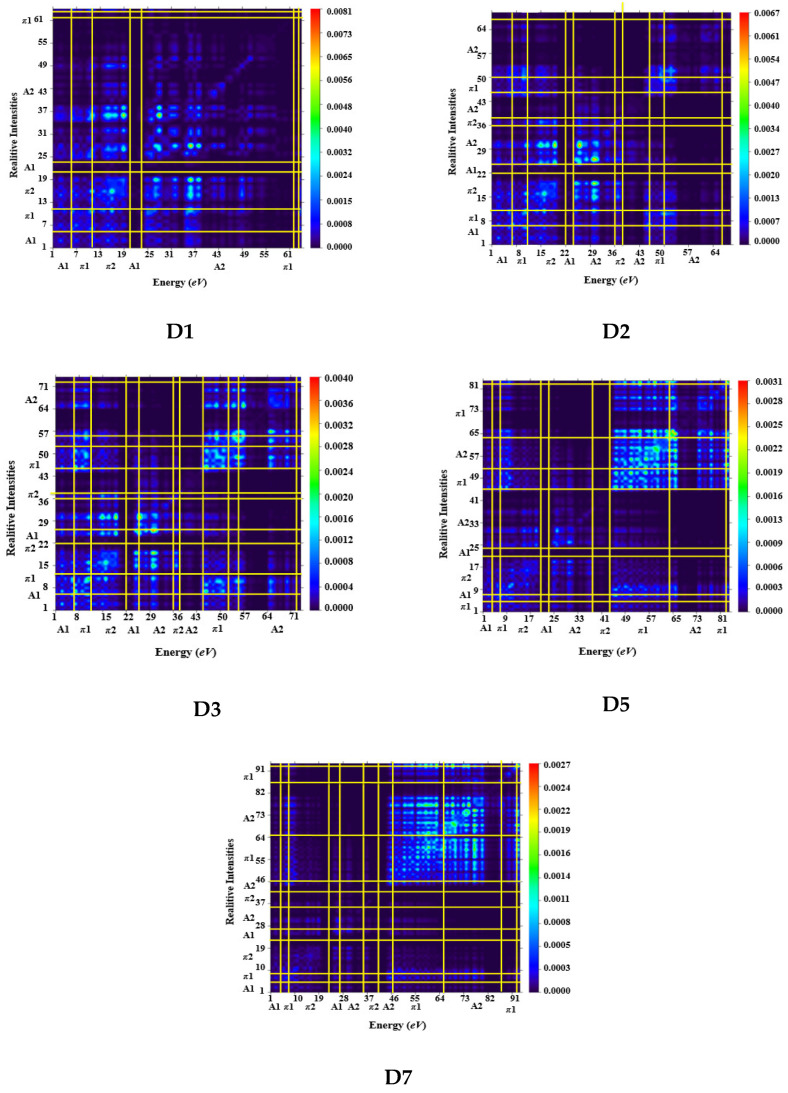
TDM graphs of the **D1**, **D2, D3, D5** and **D7** at the S1 states.

The interpretation of the transition process in a conjugated system can be effectively determined by utilizing TDM analysis [81,82]. The TDM investigation presents a three-dimensional heat map for transition among two eigenstates. It depicts the scattering of electrons as well as hole pairs and permits to analyze their coherence lengths and delocalization [83,84]. The pictorial representation of interaction among acceptor and donor entities in the S1 (excited) state is represented by the blue region in the spatial map [42,85]. The emission and absorption of studied molecules, i.e., **D1**, **D2, D3, D5** and **D7** were examined at TD-DFT/MPW1PW91 functional and 6–311 G (d,p) basis set. The effect of a hydrogen atom is ignored owing to its minor involvement in an electronic transition. The pictographs of TDM are displayed in Figure 6 with different fragments on the left side and bottom, whereas electron density is reported on the y-axis.

The uniform dissemination of electrons over the molecule diagonal transfer can be viewed from the bright portion of TDM graphs for all the computed molecules (**D1**, **D2, D3, D5** and **D7**). Moreover, electron-hole pair generation and charge coherence also appeared to proliferate non-diagonally. FMO findings revealed that the charge density is considered observed over the molecule, which causes notable variation in TDM plots. Figure 6 displayed that the electron density effectually transfers from the core to terminal acceptors through *π*-spacers in **D1**, **D2, D3, D5** and **D7** allowing efficient charge transfer.

Binding energy (*E_b_*) is another significant factor to estimate the photovoltaic response of the examined molecules. A lesser *E_b_* value results in a greater exciton dissociation in the S1 state due to less coulomb’s force between the electron and hole. The *E_b_* of **D1**, **D2, D3, D5** and **D7** are calculated from the energy of optimization (*E_opt_*) and the HOMO-LUMO energy gap (*E_gap_*) [86] as shown in Equation (8) and the computed outcomes are listed in Table 5.

*E_b_* = *E_L-H_* − *E_opt_* (8)

According to the outcomes collected in Table 5, an almost similar trend to the FMOs energy gap is noticed in the first singlet exciton energy, i.e., it decreases gradually from **D1**, **D2, D3, D5** and **D7**. Moreover, the values of *E_b_* for the titled compounds are obtained to be 0.526, 0.508, 0.475, 0.418 and 0.362 eV, respectively. The least value of *E_b_* (0.3620 eV) is investigated in **D7**, among all the designed chromophores, which illustrates that it has the highest capacity of exciton dissociation and enhanced current charge density (*J_sc_*). The decreasing order of *E_b_* is obtained as follows: **D1** > **D2** > **D3** > **D5** > **D7**. Interestingly, all the studied chromophores showed lower *E_b_* values than that of **D1** and might be used for photovoltaic applications.

### 3.6. Dipole Moment (µ_tot_) Analysis

The dipole moment (*µ*_tot_) of a molecule is directly influenced by electronegativity (E.N) difference, the greater the E.N difference, the greater would be the dipole moment (*µ*_tot_) [72]. The dipole moment values of **D1**, **D2, D3, D5** and **D7** in x, y and z directions are calculated and collected in Table 6**.**

The data from the above table illustrate that **D3** depicted the largest value of *µ*_tot_ (9.9682 *D*) of all the entitled chromophores. Overall, the decreasing order of *µ*_tot_ is as follows: **D3** > **D2** > **D5** > **D7** > **D1.** The superior *µ*_tot_ values of the entitled compounds exploited the greater polarizability in them, which indicates the higher charge transference, resulting in effective photovoltaic responses.

### 3.7. The Open-Circuit Voltage (Voc) Investigations

The open-circuit voltage (*Voc*) is an interesting approach that plays an important role in determining the performance of the OSCs [87,88]. In fact, it explains the maximum current that may be achieved from an optical substance [89]. The following influential factors affecting the *Voc* are found: light intensity, light source, external fluorescence proficiency, OSCs device’s temperature, charge carrier recombination and various other environmental elements. The *Voc* is closely related to the energy difference between HOMO and LUMO of the donor (D) and acceptor (A) compounds [90]. In order to attain a higher *Voc*, in the acceptor molecule, the LUMO level should be lower and for the donor molecule, the HOMO level should be with a high energy level [91]. Equation (9) is used to calculate the *Voc* for the designed materials, as provided by Scharber and his coworkers [92].

(9) $$V_{oc}=\left(\left|E_{HOMO}^{D}\right|-\left|E_{LUMO}^{A}\right|\right)-0.3$$

**Figure 7:**
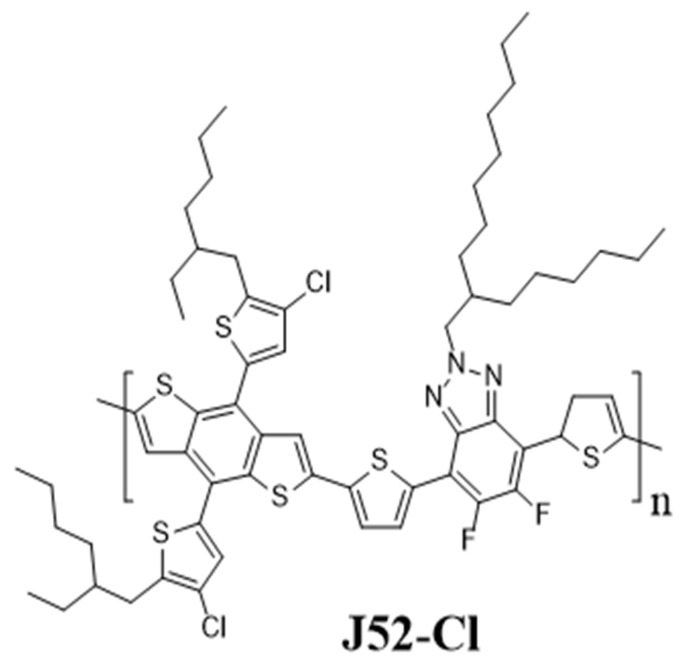
The structural representation of chlorinated polymer **J52-Cl**.

Hence, E is an elementary charge of acceptors, signifies the charge on each molecule, and 0.3 denotes the empirical constant. The chlorinated polymer **J52-Cl** is a well-known donor polymer widely used in large published reports to blend with acceptor molecules in charge transfer analysis. [93,94,95,96]. Therefore, following the literature, the studied molecules are blended with **J52-Cl** polymer to predict the potential usage of designed compounds regarding charge transfer characteristics for organic solar cells. The structural representation of **J52-Cl** is shown in Figure 7. To determine the *Voc* of the current investigation a donor polymer (**J52-Cl**) is utilized. In Table 7, the calculated values of *Voc* are illustrated along with the *E*_LUMO_ of **D1**, **D2, D3, D5** and **D7** in relation to the *E*_HOMO_ of the donor polymer (**J52-Cl**).

$$\Delta E=E_{\mathrm{LUMO}}^{A}-E_{HOMO}^{D}$$

**Figure 8:**
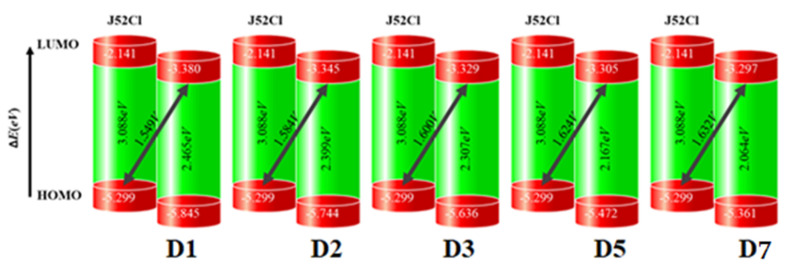
The *Voc* diagram of **D1**, **D2**, **D3**, **D5** and **D7** with respect to donor **JCl52**.

The *Voc* value for **D1**, **D2, D3, D5** and **D7** with respect to LUMO_acceptor_ HOMO_donor_ energy difference is determined to be 1.549, 1.584, 1.600, 1.624 and 1.632*V*, respectively. The *V_oc_* of entitled compounds decreases in the following order: **D7** > **D5** > **D3** > **D2** > **D1**. Among all tailored molecules, **D7** displayed the highest *Voc* (1.632 V). Since the transference of electrons from donor (D) to acceptor (A) segments, the HOMO/LUMO energy gap is a crucial tool for improving the PCEs of solar cells. A low-lying LUMO lead to improved *Voc* values having better optoelectronic properties. Open-circuit voltage (*Voc*) diagram is illustrated in Figure 8. This form of molecular orbital alignment makes it easier for the electron density to move from the donor polymer to the acceptor, as all our derivatives possess a lower value of LUMO than the **J52Cl,** which improves optoelectronic behavior.

## 4. Conclusions

The organic-based materials (**D1**, **D2, D3, D5** and **D7**) have been designed through the incorporation of selenophene units in the reference compound (**DF-PCIC**) up to n = 7. In order to improve the electron-withdrawing effect of terminal acceptors, benzothiophene-based acceptors are also introduced in **D1**, **D2, D3, D5** and **D7** compounds. After the addition of selenophene units, diminishing in band gaps (*∆E* = 2.399 − 2.064 eV) accompanied by larger bathochromic shift (λ_max_ = 655.480 − 728.376 nm) and lower binding energies (E_b_ = 0.508 − 0.362 eV) are obtained, and the conjugation is also enhanced. These findings enclosed higher exciton dissociation rate and significant charge transference from HOMO to LUMO, which is further supported by TDM and DOS analyses. The GRP studies and diminishing band gaps revealed that increasing conjugation grants significant stability to the computed chromophores. An efficient value of *V_oc_* is noticed for all POSCs materials when determined with respect to **JCl52** polymer. Among all the compounds, **D7** exhibited a lower bandgap (2.064 eV) and highest *λ_max_* (691.953 nm in gas and 728.376 nm in chloroform) and greater open-circuit voltage value (1.632 V), which proves that it is a most suitable chromophore with outstanding photovoltaic characteristics. Consequently, significant photovoltaic materials can be developed by structural tailoring with selenophene units and efficient electron-withdrawing moieties. Moreover, this study also encourages the experimentalist to synthesize these efficient materials.

## Figures and Tables

**Table 1:** *E_LUMO_*, *E_HOMO_* and energy gaps (*E_gap_* = *E_LUMO_* − *E_HOMO_*) of **D1, D2, D3, D5** and **D7**).

Chromophores	*E_HOMO_*	*E_LUMO_*	$E_{gap}$
**D1**	−5.845	−3.380	2.465
**D2**	−5.744	−3.345	2.399
**D3**	−5.636	−3.329	2.307
**D5**	−5.472	−3.305	2.167
**D7**	−5.361	−3.297	2.064

Units in eV.

**Table 2:** The calculated transition energies (eV), maximum absorption wavelengths (*λ*_max_), oscillator strengths (*f*_os_) and transition natures of **D1**, **D2, D3, D5** and **D7** in gas phase.

Compounds	DFT *λ* (nm)	*E* (eV)	*f_os_*	MO Contributions
**D1**	596.393	2.078	1.872	H→L (91%)
**D2**	613.722	2.020	2.750	H→L (90%)
**D3**	635.490	1.951	3.075	H→L (88%)
**D5**	668.702	1.854	3.524	H→L (84%)
**D7**	691.953	1.791	3.708	H→L (77%)

MO = molecular orbital, *f_os_ **=*** oscillator strength, H = HOMO, L = LUMO.

**Table 3:** Computed absorption properties of **D1**, **D2, D3, D5** and **D7** in chloroform solvent.

Compounds	DFT *λ*(nm)	*E* (eV)	*f_os_*	MO Contributions
**D1**	639.390	1.939	1.879	H→L (86%)
**D2**	655.480	1.891	2.674	H→L (85%)
**D3**	676.474	1.832	3.0738	H→L (82%)
**D5**	708.521	1.749	3.395	H→L (80%)
**D7**	728.376	1.702	3.409	H→L (75%)

**Table 4:** Global reactivity parameters of **D1**, **D2, D3, D5** and **D7**.

Compounds	*IP*	*EA*	*X*	*η*	*μ*	*ω*	*σ*
**D1**	5.845	3.380	4.613	1.233	−4.613	8.631	0.406
**D2**	5.744	3.345	4.545	1.199	−4.545	8.609	0.417
**D3**	5.636	3.329	4.483	1.154	−4.483	8.709	0.434
**D5**	5.472	3.305	4.389	1.084	−4.389	8.888	0.461
**D7**	5.631	3.297	4.464	1.167	−4.464	8.538	0.428

Units in eV.

**Table 5:** Computed binding energies (*E_b_*) of **D1**, **D2, D3, D5** and **D7**.

Chromophores	*E*_H-L_	*E_opt_*	*E_b_*
**D1**	2.465	1.939	0.526
**D2**	2.399	1.891	0.508
**D3**	2.307	1.832	0.475
**D5**	2.167	1.749	0.418
**D7**	2.064	1.702	0.362

Units in eV.

**Table 6:** Computed dipole moment (*µ*_tot_) of **D1**, **D2, D3, D5** and **D7,** *µ* units in *D*.

Chromophores	*µ*_x_	*µ*_y_	*µ*_z_	*µ*_total_
**D1**	0.7967	1.9193	−0.6419	2.1750
**D2**	3.8529	5.4304	5.1333	8.4074
**D3**	−1.5910	8.3120	−5.2673	9.9682
**D5**	−3.8684	−5.9795	1.6921	7.3200
**D7**	−1.7431	3.5215	−3.9342	5.5604

**Table 7:** Computed *Voc* of D1, D2, D3, D5 and D7.

Chromophores	*V_OC_* (V)	ΔE
**D1**	1.549	1.849
**D2**	1.584	1.884
**D3**	1.600	1.900
**D5**	1.624	1.924
**D7**	1.632	1.932

## Data Availability

All data generated or analyzed during this study are included in this published article and its Supplementary Information Files.

## Supplementary Materials

The following are available online at https://www.mdpi.com/article/10.3390/polym15061508/s1. It contain cartesian co-ordinates, molecular orbital energies, UV-Vis absorption values, FMOs diagrams showed movement of charges transference between orbitals and ICUPAC names of studied selenophene based compounds.
