# Supplementary material for: Enhancing the Photovoltaic Properties via Incorporation of Selenophene Units in Organic Chromophores with A2-π2-A1-π1-A2 Configuration: A DFT-Based Exploration
Source: Polymers (Basel). 2023 Mar 17;15(6):1508. doi: 10.3390/polym15061508 (PMC10051165; doi:10.3390/polym15061508)
Supplement: Supplementary file 1 [file polymers-15-01508-s001.zip › polymers-2187560-supplementary.pdf]

Enhancing the Photovoltaic Properties *via* Incorporation of Selenophene Units in Organic Chromophores with A_2-π_2-A_1-π_1- A_2 Configuration: A DFT-Based Exploration

Muhammad Nadeem Arshad, Iqra Shafiq, Muhammad Khalid, Mohammad Asad, Abdullah M. Asiri, Maha M. Alotaibi, Ataualpa A. C. Braga, Anish Khan and Khalid A. Alamry

Table S1: Cartesian coordinates of **D1**

Atom	X-axis	Y-axis	Z-axis
C	1.867896	-2.07665	-0.41486
C	0.619812	-1.5064	-0.39757
C	0.393971	-0.12583	-0.31853
C	1.552774	0.66114	-0.24653
C	2.800563	0.089609	-0.26683
C	3.027035	-1.28881	-0.35394
C	4.347388	-1.89236	-0.37799
C	4.625927	-3.23486	-0.54065
C	5.983113	-3.57825	-0.54866
H	3.852513	-3.97846	-0.67419
C	6.872454	-2.53849	-0.35462
C	-0.9231	0.48594	-0.31584
C	-1.19612	1.842971	-0.39035
S	-2.40123	-0.44938	-0.18672
C	-2.56509	2.124423	-0.33907
H	-0.42495	2.593731	-0.48704
C	-3.33774	0.981038	-0.22566
C	-4.71313	1.35757	-0.18359
C	-4.80512	2.742909	-0.27757
C	-6.12303	3.187056	-0.27145
C	-7.04625	2.149119	-0.13912
H	-6.4402	4.218204	-0.35495
C	-3.42771	3.372401	-0.38139
C	-3.14076	4.291489	0.814609
H	-2.1175	4.669732	0.764646
H	-3.82018	5.146213	0.806535
H	-3.26747	3.758099	1.75801
C	-3.25345	4.13122	-1.70423
H	-3.93641	4.982252	-1.7453

H	-2.23322	4.510655	-1.79192
H	-3.45625	3.482973	-2.55828
F	-0.43872	-2.33386	-0.45769
F	3.859858	0.916218	-0.19267
H	1.923695	-3.1542	-0.47354
H	1.496375	1.737018	-0.16404
C	8.287547	-2.69129	-0.42874
C	9.324208	-1.86784	-0.13117
H	8.603686	-3.616	-0.91046
C	-8.45076	2.32535	-0.19161
C	-9.50681	1.489811	0.017401
H	-8.75115	3.300051	-0.57345
C	10.60363	0.092736	0.001787
C	11.3414	-0.84166	-0.69087
C	-10.8441	-0.43802	-0.01101
C	-11.5523	0.571557	-0.62128
C	-10.8163	1.843072	-0.60281
C	10.64883	-2.13347	-0.77287
C	9.444393	-0.59586	0.578863
C	-9.65833	0.166943	0.611083
C	8.793867	-0.2986	1.750571
C	-9.00649	-0.25545	1.745179
O	11.02253	-3.15767	-1.29517
O	-11.1602	2.916262	-1.04421
C	-9.25673	-1.48251	2.412926
N	-9.41537	-2.45833	3.009578
C	-8.14479	0.609659	2.474337
N	-7.48349	1.295554	3.126384
C	7.955847	-1.2465	2.401304
N	7.313353	-2.00184	2.992127
C	9.026727	0.866795	2.527855
N	9.175013	1.786679	3.209416
S	-6.23516	0.592978	-0.06901
S	12.81375	-0.26506	-1.32946
S	-13.0455	0.098172	-1.29639
C	-11.9293	-4.07693	-0.05019
C	-11.102	-3.00762	0.226294
C	-11.5023	-1.70914	-0.12816
C	-12.7377	-1.55421	-0.80965

C	-13.5745	-2.63341	-1.07961
C	-13.1626	-3.8934	-0.68817
H	-11.6167	-5.07546	0.228323
H	-10.1526	-3.17576	0.711158
H	-14.5162	-2.48935	-1.59354
H	-13.7955	-4.74841	-0.89084
C	12.45349	1.331806	-0.7107
C	13.25176	2.457109	-0.89527
C	12.79939	3.666305	-0.40147
C	11.56458	3.756032	0.253849
C	10.77557	2.640465	0.445742
C	11.21809	1.390032	-0.01478
H	14.19468	2.387888	-1.42221
H	13.40164	4.556064	-0.53649
H	11.22083	4.717676	0.613403
H	9.82386	2.735534	0.945938
Se	5.93929	-0.91758	-0.1928
H	6.326554	-4.59474	-0.69554

Table S2: Cartesian coordinates of **D2**.

Atom	X-axis	Y-axis	Z-axis
C	-0.29484	-0.79118	-0.32523
C	-1.61975	-0.43204	-0.32043
C	-2.06951	0.894012	-0.35246
C	-1.05439	1.861389	-0.38348
C	0.26998	1.500771	-0.39119
C	0.72067	0.175945	-0.36508
C	2.122357	-0.20276	-0.37476
C	2.623252	-1.48584	-0.39053
C	4.02269	-1.60464	-0.39208
H	1.985598	-2.35928	-0.4046
C	4.725146	-0.4211	-0.37774
C	-3.46947	1.281922	-0.3611
C	-3.96299	2.565732	-0.53251
S	-4.77191	0.131919	-0.12015
C	-5.35905	2.623849	-0.46629
H	-3.32735	3.42207	-0.70666
C	-5.93197	1.383235	-0.24465

C	-7.34947	1.53366	-0.19433
C	-7.66917	2.873976	-0.39144
C	-9.04146	3.097293	-0.38297
C	-9.78061	1.937036	-0.14655
H	-9.52458	4.053375	-0.53615
C	-6.41497	3.70797	-0.58082
C	-6.26495	4.754497	0.532549
H	-5.31786	5.287598	0.425805
H	-7.07458	5.485049	0.477069
H	-6.28958	4.285276	1.517344
C	-6.38638	4.376232	-1.96233
H	-7.19989	5.099357	-2.04997
H	-5.44329	4.907434	-2.10694
H	-6.49283	3.637173	-2.75808
F	-2.52807	-1.42382	-0.28014
F	1.179755	2.493765	-0.41934
H	-0.06442	-1.84659	-0.29616
H	-1.28508	2.916992	-0.39392
C	-11.195	1.878163	-0.17355
C	-12.0968	0.899823	0.123562
H	-11.657	2.759328	-0.61651
C	-13.0982	-1.21734	0.270563
C	-13.9715	-0.38612	-0.39225
C	-13.4546	0.986294	-0.48599
C	-12.0193	-0.38092	0.814912
C	-11.2892	-0.60369	1.958357
O	-13.9773	1.951914	-0.99536
C	-11.3181	-1.80238	2.717512
N	-11.2997	-2.7442	3.385449
C	-10.575	0.444975	2.600954
N	-10.0298	1.278459	3.184892
S	-8.72378	0.543922	0.01993
S	-15.3759	-1.14633	-0.99148
C	-13.5713	-4.97694	0.52446
C	-12.9272	-3.76933	0.70091
C	-13.54	-2.58395	0.263564
C	-14.7934	-2.68461	-0.39482
C	-15.4452	-3.90296	-0.56398
C	-14.8266	-5.04576	-0.09304

H	-13.095	-5.88714	0.866546
H	-11.9565	-3.74338	1.171808
H	-16.4045	-3.9532	-1.06262
H	-15.3131	-6.00531	-0.21677
Se	3.53726	1.03473	-0.36166
H	4.513034	-2.5706	-0.40654
C	6.145281	-0.23236	-0.37637
C	6.842175	0.965177	-0.38825
C	8.235956	0.872079	-0.38023
H	6.334654	1.922013	-0.40846
C	8.752457	-0.41384	-0.35868
H	8.889246	1.731737	-0.39105
Se	7.338632	-1.66968	-0.35844
C	10.07385	-0.93421	-0.36598
C	11.30353	-0.34494	-0.25168
H	10.09971	-2.01506	-0.44406
C	12.58032	-1.07451	-0.27184
C	11.61857	1.070518	0.068818
C	15.32471	1.05524	1.279755
C	14.9265	-0.24536	0.871892
C	15.81806	-1.30451	1.107217
C	17.05424	-1.05042	1.666371
C	17.44017	0.249108	2.015549
C	16.57555	1.311734	1.833352
H	15.54591	-2.31808	0.859508
H	17.73506	-1.874	1.841175
H	18.41744	0.423185	2.448255
H	16.85568	2.315399	2.126305
C	13.0334	1.049045	0.454163
C	13.5831	-0.20577	0.354235
S	14.06788	2.254072	1.067667
O	10.89557	2.044823	0.057956
C	12.81212	-2.26272	-0.92314
C	11.80839	-2.97528	-1.63788
N	11.04332	-3.59553	-2.24121
C	14.091	-2.85748	-1.08749
N	15.09451	-3.39107	-1.29173

Table S3: Cartesian coordinates of **D3**.

Atom	X-axis	Y-axis	Z-axis
C	2.241293	-0.89414	-0.03166
C	3.556343	-0.50419	0.022683
C	3.972409	0.827252	0.147051
C	2.931842	1.765666	0.213061
C	1.617172	1.374155	0.160727
C	1.200329	0.043898	0.034686
C	-0.19032	-0.36825	-0.03026
C	-0.65621	-1.65235	-0.19584
C	-2.0536	-1.80808	-0.2279
H	0.005225	-2.50119	-0.30602
C	-2.78849	-0.65403	-0.08968
C	5.362123	1.246189	0.212121
C	5.822411	2.536441	0.424199
S	6.696816	0.127276	0.001745
C	7.218693	2.623372	0.413907
H	5.163523	3.377781	0.584444
C	7.825125	1.398516	0.194427
C	9.239689	1.577518	0.200255
C	9.524788	2.92053	0.43089
C	10.89141	3.170141	0.477948
C	11.66242	2.028278	0.251819
H	11.34977	4.132418	0.664644
C	8.247339	3.726201	0.585752
C	8.118231	4.78824	-0.51541
H	7.157098	5.300655	-0.4365
H	8.91011	5.533632	-0.41717
H	8.189769	4.336361	-1.50602
C	8.152679	4.370238	1.975825
H	8.94644	5.108747	2.105967
H	7.193462	4.878842	2.093354
H	8.245176	3.620099	2.762909
F	4.489424	-1.47151	-0.04846
F	0.682065	2.341835	0.231983
H	2.038534	-1.95156	-0.12412
H	3.134416	2.823428	0.300819
C	13.07498	1.997606	0.334034
C	14.00877	1.040813	0.065106
H	13.50022	2.883629	0.803382
C	15.0595	-1.05375	-0.05938

C	15.88734	-0.21149	0.646121
C	15.33797	1.149028	0.731148
C	13.98665	-0.23402	-0.64027
C	13.30927	-0.46096	-1.81507
O	15.81898	2.119822	1.271054
C	13.39635	-1.6509	-2.58347
N	13.42665	-2.586	-3.26038
C	12.60071	0.57889	-2.47776
N	12.06293	1.406625	-3.07665
S	10.64017	0.617832	0.022842
S	17.2814	-0.94889	1.296157
C	15.61848	-4.80062	-0.32519
C	14.95807	-3.6044	-0.51802
C	15.52837	-2.41121	-0.04586
C	16.75549	-2.49299	0.662806
C	17.42408	-3.69978	0.848507
C	16.84851	-4.85024	0.342986
H	15.17509	-5.71691	-0.69421
H	14.00721	-3.59337	-1.0284
H	18.363	-3.73581	1.385838
H	17.34866	-5.80107	0.478743
Se	-1.64253	0.821135	0.112691
H	-2.51784	-2.77637	-0.37104
C	-4.21513	-0.50434	-0.08726
C	-4.95464	0.654143	-0.17202
C	-6.35099	0.495184	-0.15048
H	-4.48937	1.627252	-0.27036
C	-6.81141	-0.79835	-0.04795
H	-7.02443	1.340038	-0.22961
Se	-5.35595	-1.99288	0.05205
C	-8.1649	-1.25977	-0.01258
C	-8.60501	-2.57271	-0.05788
C	-9.98763	-2.73412	-0.00978
H	-7.92657	-3.41335	-0.13531
C	-10.7507	-1.57363	0.075884
H	-10.4622	-3.70766	-0.03706
Se	-9.61252	-0.06639	0.110441
C	-12.1628	-1.65198	0.140531
C	-13.1558	-0.71247	0.189704

H	-12.4879	-2.68596	0.119141
C	-16.4951	2.245232	-0.55457
C	-16.5643	0.833041	-0.4189
C	-17.7921	0.21249	-0.70257
C	-18.8881	0.979066	-1.04345
C	-18.8033	2.374126	-1.12222
C	-17.6038	3.018268	-0.8869
H	-17.8892	-0.86037	-0.6619
H	-19.8296	0.489071	-1.25762
H	-19.679	2.95368	-1.38664
H	-17.5215	4.094074	-0.97328
C	-12.9643	0.74652	0.117565
C	-14.6003	-0.96329	0.239272
C	-14.3099	1.29649	-0.05377
C	-15.2649	0.308577	-0.08284
S	-14.8803	2.877106	-0.32557
O	-11.9202	1.369287	0.160886
C	-15.2076	-2.12125	0.66597
C	-14.4925	-3.28988	1.049721
N	-13.9706	-4.2673	1.376111
C	-16.5998	-2.26555	0.903476
N	-17.7103	-2.45944	1.154391

Table S4: Cartesian coordinates of **D5**.

Atom	X-axis	Y-axis	Z-axis
C	-6.2279	-0.51375	0.651398
C	-7.55628	-0.18756	0.53432
C	-8.01862	1.072745	0.136412
C	-7.01245	2.006074	-0.15252
C	-5.68449	1.679344	-0.03216
C	-5.22047	0.422056	0.37201
C	-3.81569	0.0774	0.497669
C	-3.30248	-1.13252	0.902708
C	-1.89926	-1.22976	0.95361
H	-3.93243	-1.96769	1.178522
C	-1.20695	-0.09859	0.591901
C	-9.42249	1.431806	0.028405
C	-9.93017	2.703913	-0.18295

S	-10.7107	0.245928	0.126698
C	-11.3265	2.722776	-0.27198
H	-9.30422	3.581736	-0.25704
C	-11.8851	1.464656	-0.12647
C	-13.304	1.578586	-0.21188
C	-13.6389	2.913229	-0.42438
C	-15.0104	3.100686	-0.5502
C	-15.7382	1.91827	-0.40251
H	-15.5023	4.04602	-0.73823
C	-12.3946	3.781369	-0.47686
C	-12.3841	4.814328	0.658841
H	-11.4454	5.372255	0.654658
H	-13.203	5.525373	0.531239
H	-12.4951	4.329846	1.63021
C	-12.2455	4.470981	-1.84013
H	-13.0636	5.176421	-1.99955
H	-11.3056	5.025519	-1.88318
H	-12.2541	3.741601	-2.65175
F	-8.45523	-1.14719	0.821739
F	-4.78437	2.636445	-0.3329
H	-5.98726	-1.51845	0.968595
H	-7.25394	3.004566	-0.48806
C	-17.1403	1.825298	-0.56863
C	-18.0413	0.818908	-0.37988
H	-17.5794	2.704172	-1.03854
C	-18.9965	-1.32438	-0.36975
C	-19.8228	-0.50162	-1.09951
C	-19.3341	0.884478	-1.11875
C	-17.9985	-0.4727	0.292949
C	-17.3802	-0.70138	1.49963
O	-19.8293	1.846829	-1.6611
C	-17.455	-1.9155	2.230557
N	-17.4803	-2.86992	2.880173
C	-16.7619	0.350964	2.229544
N	-16.3006	1.18568	2.880251
S	-14.6653	0.550148	-0.14944
S	-21.1419	-1.28414	-1.84625
C	-19.3886	-5.09906	-0.23316
C	-18.7978	-3.87946	0.027116

C	-19.3983	-2.70112	-0.44495
C	-20.5791	-2.81951	-1.22353
C	-21.1781	-4.05022	-1.47726
C	-20.5768	-5.1866	-0.96982
H	-18.9226	-6.00394	0.13608
H	-17.8774	-3.83859	0.589334
H	-22.0835	-4.11444	-2.06703
H	-21.0228	-6.15535	-1.1575
Se	-2.4057570	1.26679	0.114049
H	-1.39968	-2.135	1.276948
C	0.213875	0.100719	0.546749
C	0.912521	1.279539	0.446032
C	2.317271	1.16664	0.432328
H	0.414305	2.240658	0.405689
C	2.820721	-0.10887	0.521438
H	2.961825	2.035925	0.379921
Se	1.40945400	-1.35406	0.614204
C	4.190848	-0.53112	0.539086
C	4.704553	-1.76562	0.859601
C	6.105951	-1.88734	0.793563
H	4.071146	-2.58845	1.168096
C	6.79297	-0.7579	0.41666
H	6.61337	-2.80991	1.048811
Se	5.58425000	0.647082	0.072705
C	8.206942	-0.57787	0.272748
C	8.909432	0.597658	0.122432
C	10.3037	0.469816	0.006745
H	8.41876	1.563228	0.112748
C	10.80175	-0.81322	0.055618
H	10.94907	1.332763	-0.10467
C	12.16342	-1.24081	-0.02552
C	12.64006	-2.54068	0.039723
C	14.02256	-2.66943	-0.06415
H	11.98738	-3.39632	0.161547
C	14.75171	-1.49365	-0.21633
H	14.52229	-3.63017	-0.03296
C	16.16087	-1.53886	-0.33782
C	17.12701	-0.57645	-0.45173
H	16.51357	-2.56333	-0.30104

C	18.57413	-0.79262	-0.55094
C	16.90064	0.878442	-0.41098
C	20.41462	2.483381	0.079756
C	20.51497	1.070021	-0.01965
C	21.76812	0.488287	0.233535
C	22.85623	1.291122	0.510538
C	22.73864	2.685378	0.553918
C	21.5148	3.292763	0.347474
H	21.89106	-0.5826	0.218934
H	23.81746	0.830828	0.701703
H	23.60852	3.2936	0.768233
H	21.40799	4.368252	0.407356
C	18.23718	1.466693	-0.30914
C	19.21783	0.504169	-0.2905
S	18.77643	3.068105	-0.10333
O	15.83981	1.473789	-0.42802
C	19.19517	-1.94695	-0.96822
C	18.49724	-3.1444	-1.28962
N	17.98955	-4.14429	-1.56666
C	20.58034	-2.06202	-1.25689
N	21.68498	-2.23431	-1.54653
Se	9.38573800	-2.04349	0.259214
Se	13.57457600	-0.01584	-0.2395

Table S5: Cartesian coordinates of **D7**.

Atom	X-axis	Y-axis	Z-axis
C	10.21363	0.596513	0.029693
C	11.54017	0.268113	-0.10151
C	12.01557	-1.04706	-0.16807
C	11.02663	-2.03762	-0.08196
C	9.700084	-1.70761	0.045595
C	9.222111	-0.39339	0.108079
C	7.819298	-0.04607	0.247082
C	7.292269	1.222186	0.31663
C	5.894287	1.310973	0.455769
H	7.908759	2.110285	0.275676
C	5.22014	0.114046	0.506626
C	13.41537	-1.40335	-0.32656

C	13.91337	-2.66569	-0.60685
S	14.7131	-0.23933	-0.13812
C	15.3113	-2.69718	-0.66198
H	13.27769	-3.52323	-0.77535
C	15.88045	-1.45799	-0.42389
C	17.29884	-1.57941	-0.50831
C	17.623	-2.90014	-0.80701
C	18.99374	-3.09303	-0.93282
C	19.73157	-1.93109	-0.69853
H	19.47774	-4.0285	-1.18118
C	16.37111	-3.74996	-0.92984
C	16.34061	-4.85831	0.132189
H	15.39717	-5.4059	0.080951
H	17.1546	-5.56639	-0.03626
H	16.44617	-4.44271	1.135562
C	16.22819	-4.34311	-2.33831
H	17.04121	-5.04402	-2.53857
H	15.28385	-4.88432	-2.42739
H	16.25104	-3.56005	-3.09797
F	12.42385	1.281257	-0.16851
F	8.816963	-2.72299	0.122487
H	9.961103	1.646644	0.067512
H	11.28245	-3.08752	-0.10145
C	21.13578	-1.83891	-0.84664
C	22.0426	-0.85773	-0.57224
H	21.57195	-2.68242	-1.37982
C	23.01636	1.268518	-0.37798
C	23.84116	0.50106	-1.16723
C	23.34091	-0.87428	-1.30397
C	22.00452	0.374889	0.203641
C	21.37591	0.510008	1.419104
O	23.8326	-1.7935	-1.91944
C	21.45491	1.658661	2.248621
N	21.48246	2.555659	2.97541
C	20.7388	-0.59156	2.053961
N	20.26118	-1.47099	2.629752
S	18.66967	-0.57242	-0.3618
S	25.17305	1.330003	-1.83735
C	23.44248	5.014966	0.074438

C	22.83852	3.783839	0.228708
C	23.43132	2.642818	-0.33575
C	24.61915	2.813858	-1.09368
C	25.23124	4.055436	-1.24058
C	24.63663	5.151735	-0.64476
H	22.98217	5.890595	0.514582
H	21.914	3.705604	0.780077
H	26.14132	4.159319	-1.81723
H	25.09287	6.128328	-0.74869
Se	6.430481	-1.31508	0.3553
H	5.386576	2.264657	0.535467
C	3.808943	-0.11044	0.643118
C	3.151093	-1.27864	0.942094
C	1.745743	-1.19624	1.017979
H	3.681853	-2.20357	1.133218
C	1.201043	0.043235	0.78445
H	1.133558	-2.05337	1.271418
Se	2.565168	1.284293	0.397177
C	-0.17939	0.432551	0.797339
C	-0.71513	1.697236	0.828287
C	-2.12281	1.773036	0.840863
H	-0.0924	2.582882	0.867422
C	-2.79183	0.573202	0.820465
H	-2.64538	2.720813	0.890119
Se	-1.55873	-0.85104	0.754625
C	-4.20582	0.334124	0.8325
C	-4.87583	-0.84293	1.065604
C	-6.28263	-0.77383	1.018169
H	-4.35455	-1.76392	1.29742
C	-6.81729	0.461921	0.744329
H	-6.90589	-1.63859	1.211909
C	-8.19672	0.838061	0.639718
C	-8.74319	2.099739	0.618898
C	-10.1458	2.163373	0.511447
H	-8.13305	2.990677	0.70462
C	-10.8016	0.957277	0.439603
H	-10.6773	3.107483	0.508747
Se	-5.43635	1.718497	0.484603
Se	-9.55596	-0.4571	0.488738

C	-12.2084	0.709142	0.333487
C	-12.8789	-0.48737	0.464143
C	-14.2739	-0.43091	0.307017
H	-12.3641	-1.41264	0.692272
C	-14.8045	0.811784	0.041416
H	-14.8964	-1.31205	0.405316
C	-16.174	1.169261	-0.15832
C	-16.6939	2.444903	-0.31211
C	-18.0732	2.503178	-0.49185
H	-16.0751	3.333436	-0.28777
C	-18.7567	1.290515	-0.49508
H	-18.6046	3.437784	-0.6254
C	-20.1582	1.262452	-0.68766
H	-20.5445	2.266534	-0.82132
C	-21.0858	0.256342	-0.70579
C	-20.8156	-1.16662	-0.43863
C	-22.5306	0.397014	-0.91456
C	-22.1361	-1.78801	-0.32575
C	-23.1469	-0.87436	-0.50469
C	-23.1623	1.453752	-1.52833
C	-24.4393	-1.44884	-0.2286
C	-25.7249	-0.89025	-0.13692
C	-26.801	-1.68972	0.19179
C	-26.6402	-3.05671	0.44679
C	-25.3862	-3.63533	0.402961
C	-24.2988	-2.82757	0.082989
H	-25.8829	0.160835	-0.31668
H	-27.7872	-1.24754	0.256945
H	-27.5011	-3.66361	0.697652
H	-25.2476	-4.68539	0.626225
S	-22.6341	-3.3637	0.082142
O	-19.7371	-1.71469	-0.30942
C	-24.5294	1.468466	-1.91038
N	-25.6184	1.551412	-2.28593
C	-22.4866	2.62242	-1.9776
N	-21.9972	3.594254	-2.36535
Se	-17.5328	-0.12782	-0.24751
Se	-13.4237	2.094711	-0.04333

Table S6: Calculated energies (*E*) and energy gap (*ΔE*) for **D1-D7**.

Compounds	*E LUMO+1*	*E HOMO-1*	*ΔE (eV)*	*E LUMO+2*	*E HOMO-2*	*ΔE (eV)*
**D1**	-3.178	-6.606	3.428	-3.032	-6.914	3.882
**D2**	-3.184	-6.262	3.078	-3.072	-6.895	3.823
**D3**	-3.204	-6.056	2.852	-3.064	-6.762	3.698
**D5**	-3.208	-5.853	2.645	-3.066	-6.289	3.223
**D7**	-3.209	-5.719	2.510	-3.065	-6.021	2.956

E= energy, Δ*E*(e*V*)= E_{LUMO}-E_{HOMO}; HOMO= highest occupied molecular orbital; LUMO= lowest unoccupied molecular orbital, MO, molecular orbital, Units in e*V*.

Table S7: Percentages of Acceptor 2, π-spacer 1, π-spacer 2 and Acceptor 1 for HOMOs and LUMOs

Compounds	LUMO				HOMO			
	**Acceptor- 2**	**Acceptor- 1**	**Spacer - 2**	**Spacer - 1**	**Acceptor- 2**	**Acceptor- 1**	**Spacer - 2**	**Spacer - 1**
**D1**	60.0	9.8	15.0	15.3	22.4	14.1	54.8	8.7
**D2**	53.4	7.1	12.7	26.9	18.5	14.7	42.2	24.6
**D3**	59.1	3.8	7.2	30.0	14.1	13.0	27.6	45.3
**D5**	61.8	1.0	2.1	35.1	7.4	7.2	10.6	74.8
**D7**	64.0	0.2	0.4	35.5	3.7	4.2	4.8	87.3

Table S8: Wave length, excitation energy and oscillator strength of investigated compound **D1** in gas phase.

NO	DFT *λ (nm)*	*E (eV)*	*f*	MO contributions
1	596.393	2.078	1.872	H→L (91%), H-1→L+1 (3%), H→L+1 (3%)
2	538.734	2.301	0.057	H→L+1 (61%), H→L+2 (17%), H→L+3 (11%), H-1→L+2 (3%), H→L (4%)
3	507.508	2.443	0.380	H→L+1 (24%), H→L+2 (57%), H-1→L+1 (5%), H-1→L+3 (2%), H→L+3 (6%)
4	467.988	2.649	0.091	H-1→L (13%), H→L+2 (11%), H→L+3 (64%), H→L+1 (6%)
5	439.613	2.820	0.057	H-1→L (61%), H-1→L+1 (10%), H→L+3 (13%), H-4→L+1 (2%), H→L+2 (6%)
6	413.625	2.998	0.019	H-1→L (18%), H-1→L+1 (45%), H-1→L+2 (12%), H-4→L+2 (2%), H-3→L (9%), H-3→L+1 (2%), H→L+1 (2%), H→L+2 (4%)

Table S9: Wave length, excitation energy and oscillator strength of investigated compound **D2** in gas phase.

NO	DFT λ (nm)	E (eV)	f	MO contributions
1	613.722	2.020	2.750	H→L (90%), H-1→L+1 (4%), HOMO→L+1 (3%)
2	550.747	2.251	0.076	H→L+1 (67%), H→L+3 (15%), H-1→L+2 (7%), H-1→L+3 (3%), H→L (3%)
3	533.977	2.322	0.265	H→L+2 (66%), H-1→L+1 (7%), H-1→L+2 (6%), H-1→L+3 (4%), H→L+1 (4%), H→L+3 (9%)
4	495.560	2.502	0.151	H-1→L (15%), H→L+1 (17%), H→L+2 (11%), H→L+3 (50%),
5	467.936	2.650	0.124	H-1→L (78%), H→L+3 (14%), H→L+2 (5%)
6	446.581	2.776	0.024	H-1→L+1 (58%), H-1→L+2 (14%), H→L+2 (14%), H→L+1 (3%)

Table S10: Wave length, excitation energy and oscillator strength of investigated compound **D3** in gas phase.

NO	DFT λ (nm)	E (eV)	f	MO contributions
1	635.490	1.951	3.075	H→L (88%), H-1→L+1 (6%), HOMO→L+1 (3%)
2	579.582	2.139	0.093	H-1→L+2 (10%), H→L+1 (69%), H-1→L (4%), H→L (2%), H→L+2 (8%), H→L+3 (3%)
3	554.367	2.237	0.284	H→L+2 (62%), H→L+3 (13%), H-1→L+1 (7%), H-1→L+3 (9%), H→L+1 (4%)
4	517.269	2.397	0.096	H-1→L (19%), H→L+1 (16%), H→L+2 (10%), H→L+3 (43%), H-1→L+3 (4%)
5	495.026	2.505	0.009	H-1→L (72%), H→L+3 (18%), H-1→L+3 (2%), H→L+2 (4%)
6	475.673	2.607	0.180	H-1→L+1 (77%), H→L (6%), H→L+2 (8%), H→L+3 (3%)

Table S11: Wave length, excitation energy and oscillator strength of investigated compound **D5** in gas phase.

NO	DFT λ (nm)	E (eV)	f	MO contributions
1	668.702	1.854	3.524	H→L (84%), H-1→L+1 (6%), H→L+1 (4%)
2	607.408	2.041	0.287	H→L+1 (63%), H→L+2 (15%), H-1→L (3%), H-1→L+2 (6%), H→L (5%)
3	582.113	2.130	0.080	H→L+1 (14%), H→L+2 (66%), H-1→L+1 (6%), H-1→L+2 (2%), H-1→L+3 (5%)
4	545.825	2.272	0.077	H-1→L (13%), H-1→L+1 (12%), H-1→L+3 (14%), H→L+1 (11%), H→L+3 (38%), H-2→L+1 (3%)
5	523.317	2.369	0.061	H-1→L (59%), H→L+3 (22%), H-1→L+1 (6%), H-1→L+3 (6%)

6	515.655	2.404	0.613	H-1→L (13%), H-1→L+1 (50%), H-2→L (5%), H→L (8%), H→L+1 (3%), H→L+2 (3%), H→L+3 (8%), H→L+4 (7%)

Table S12: Wavelength, excitation energy and oscillator strength of investigated compound **D7** in gas phase.

**NO**	**DFT** *λ (nm)*	*E (eV)*	*f*	**MO contributions**
1	691.953	1.791	3.708	H→L (77%), H-1→L (3%), H-1→L+1 (4%), H→L+1 (6%), H→L+2 (2%), H→L+4 (4%)
2	630.193	1.967	0.511	H→L (11%), H→L+1 (66%), H-1→L (2%), H-1→L+1 (6%), H-1→L+2 (3%), H→L+2 (7%)
3	603.858	2.053	0.080	H→L+1 (10%), H→L+2 (72%), H-2→L+2 (3%), H-1→L+1 (3%), H-1→L+2 (7%)
4	563.488	2.200	0.106	H-1→L (23%), H-1→L+1 (17%), H-1→L+3 (10%), H→L+3 (24%), H-2→L+1 (7%), H-2→L+3 (4%), H→L (2%), H→L+1 (5%)
5	551.678	2.247	0.687	H-1→L (32%), H-1→L+1 (25%), H→L+4 (10%), H-2→L (8%), H-2→L+1 (2%), H→L (8%), H→L+1 (7%)
6	546.258	2.270	0.400	H-1→L (21%), H-1→L+1 (14%), H→L+3 (40%), H-1→L+3 (8%), H→L+1 (5%), H→L+4 (5%)

Table S13: Wave length, excitation energy and oscillator strength of investigated compound **D1** in chloroform.

**NO**	**DFT** *λ (nm)*	*E (eV)*	*f*	**MO contributions**
1	639.390	1.939	1.879	H→L (86%), H-1→L+1 (3%), H→L+1 (8%)
2	577.288	2.148	0.155	H→L+1 (65%), H→L+2 (16%), H-1→L (3%), H-1→L+2 (3%), H→L (8%), H→L+3 (3%)
3	535.176	2.317	0.480	H→L+1 (17%), H→L+2 (67%), H-1→L+1 (5%), H-1→L+3 (2%), H→L+3 (5%)
4	484.465	2.559	0.041	H→L+3 (82%), H-1→L (3%), H-1→L+2 (3%), H→L+2 (6%)
5	460.925	2.690	0.093	H-1→L (72%), H-1→L+1 (9%), H→L (3%), H→L+1 (4%), H→L+2 (2%), H→L+3 (4%)
6	426.972	2.904	0.013	H-1→L (15%), H-1→L+1 (51%), H-1→L+2 (13%), H-3→L (4%), H→L+2 (5%)

Table S14: Wave length, excitation energy and oscillator strength of investigated compound **D2** in chloroform.

NO	DFT λ (nm)	E (eV)	f	MO contributions
1	655.480	1.891	2.674	H→L (85%), H-1→L+1 (5%), H→L+1 (8%)
2	592.659	2.092	0.265	H→L+1 (66%), H-1→L (6%), H-1→L+2 (4%), H→L (7%), H→L+2 (7%), H→L+3 (6%)
3	562.108	2.206	0.391	H→L+2 (67%), H-1→L+1 (8%), H-1→L+2 (5%), H-1→L+3 (3%), H→L+1 (8%), H→L+3 (5%)
4	516.773	2.399	0.081	H→L+3 (78%), H-1→L+2 (4%), H→L+1 (6%), H→L+2 (7%)
5	492.001	2.520	0.163	H-1→L (88%), H→L+1 (6%)
6	463.787	2.673	0.025	H-1→L+1 (65%), H→L+2 (14%), H-1→L+2 (9%), H→L (3%)

Table S15: Wave length, excitation energy and oscillator strength of investigated compound **D3** in chloroform.

NO	DFT λ (nm)	E (eV)	f	MO contributions
1	676.474	1.832	3.0738	H→L (82%), H-1→L+1 (7%), HOMO→L+1 (8%)
2	620.417	1.998	0.015	H→L+1 (72%), H-1→L (9%), H-1→L+2 (5%), H→L (7%)
3	586.602	2.114	0.574	H→L+2 (76%), H-1→L+1 (8%), H-1→L+2 (2%), H-1→L+3 (6%), H→L+3 (4%)
4	540.307	2.295	0.088	H→L+3 (75%), H-1→L+2 (6%), H-1→L+3 (5%), H→L+1 (5%), H→L+2 (6%)
5	520.221	2.383	0.022	H-1→L (82%), H→L (2%), H→L+1 (8%)
6	496.175	2.499	0.161	H-1→L+1 (74%), H-2→L (2%), H→L (8%), H→L+2 (9%)

Table S16: Wave length, excitation energy and oscillator strength of investigated compound **D5** in chloroform.

NO	DFT λ (nm)	E (eV)	f	MO contributions
1	708.521	1.749	3.395	H→L (80%), H-1→L (3%), H-1→L+1 (5%), H→L+1 (5%), H→L+2 (3%)
2	650.494	1.906	0.276	H-1→L+1 (11%), H→L+1 (68%), H-1→L (6%), H-1→L+2 (2%), H→L (7%)
3	615.673	2.014	0.329	H→L+2 (77%), H-2→L+2 (3%), H-1→L+1 (7%), H-1→L+2 (4%), H-1→L+3 (3%)
4	572.279	2.167	0.171	H-1→L+3 (14%), H→L+3 (55%), H-2→L+1 (2%), H-1→L+1 (8%), H-1→L+2 (4%), H→L+1 (7%), H→L+2 (3%)
5	552.687	2.243	0.105	H-1→L (42%), H-1→L+1 (22%), H→L+1 (14%), H→L+3 (12%), H-2→L+1 (3%), H-1→L+3 (3%)
6	539.766	2.297	0.438	H-1→L (34%), H-1→L+1 (33%), H→L (10%), H-2→L (8%), H→L+1 (3%), H→L+2 (2%), H→L+3 (4%)

Table S17: Wave length, excitation energy and oscillator strength of investigated compound **D7** in chloroform.

NO	DFT *λ (nm)*	*E (eV)*	*f*	MO contributions
1	728.376	1.702	3.409	H→L (75%), H-1→L (6%), H-1→L+1 (3%), H→L+1 (4%), H→L+2 (5%), H→L+4 (3%)
2	670.257	1.850	0.667	H-1→L+1 (15%), H→L+1 (64%), H-2→L+1 (4%), H-1→L (3%), H→L (7%)
3	633.542	1.957	0.312	H→L+2 (73%), H-2→L+2 (3%), H-1→L+1 (4%), H-1→L+2 (8%), H→L (3%)
4	591.951	2.095	0.110	H-1→L+1 (21%), H-1→L+3 (10%), H→L+3 (32%), H-2→L+1 (9%), H-2→L+3 (3%), H-1→L (4%), H→L+1 (9%), H→L+2 (2%)
5	579.122	2.141	0.044	H-1→L (47%), H→L+3 (19%), H-2→L (6%), H-1→L+1 (5%), H-1→L+3 (6%), H→L (4%), H→L+1 (7%)
6	575.520	2.154	0.961	H-1→L (19%), H-1→L+1 (27%), H→L+1 (12%), H→L+3 (13%), H-2→L (6%), H-2→L+1 (3%), H→L (9%), H→L+4 (5%)

MO=molecular orbital, HOMO=H, LUMO=L, *f* = oscillator strength

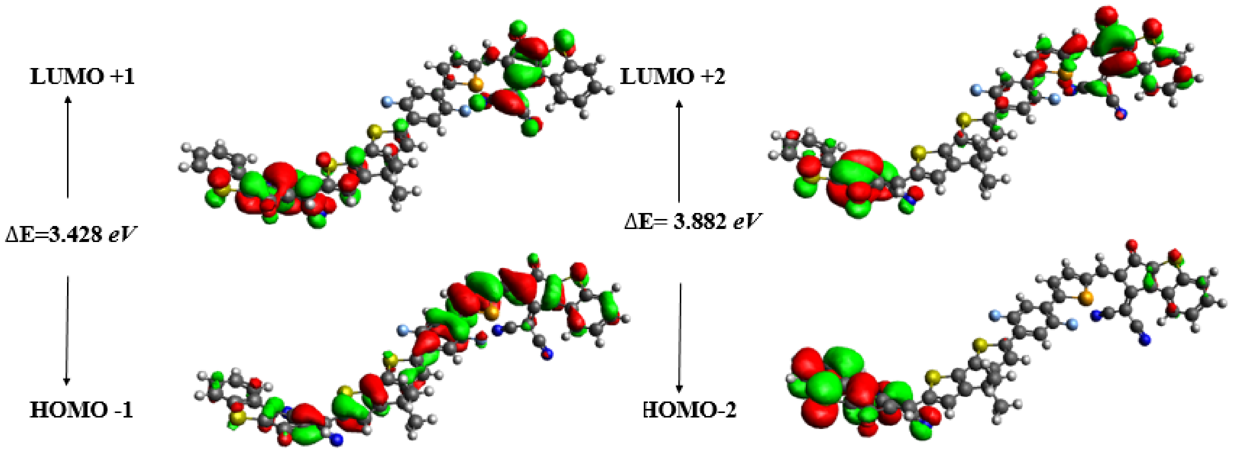

Figure S1: Frontier molecular orbitals of **D1**.

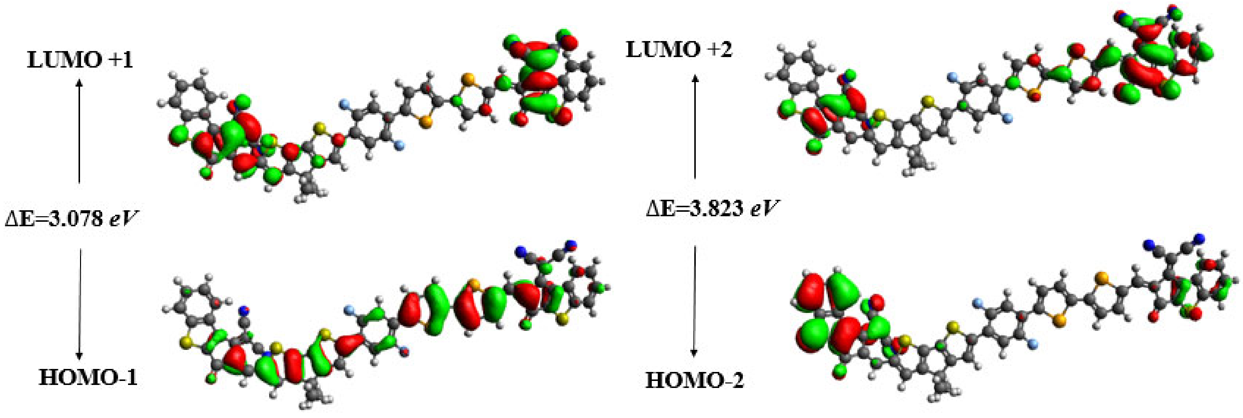

Figure S2: Frontier molecular orbitals of **D2**.

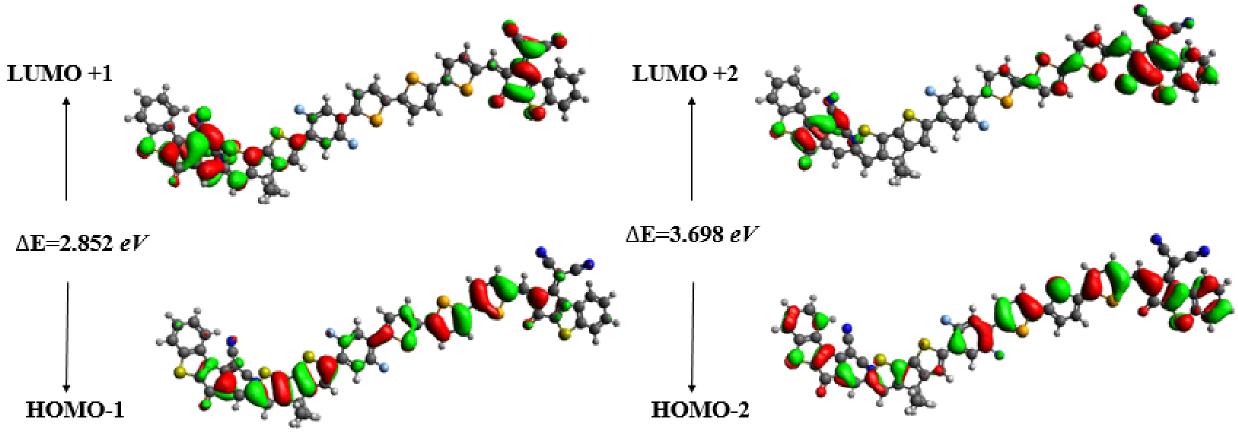

Figure S3: Frontier molecular orbitals of **D3**.

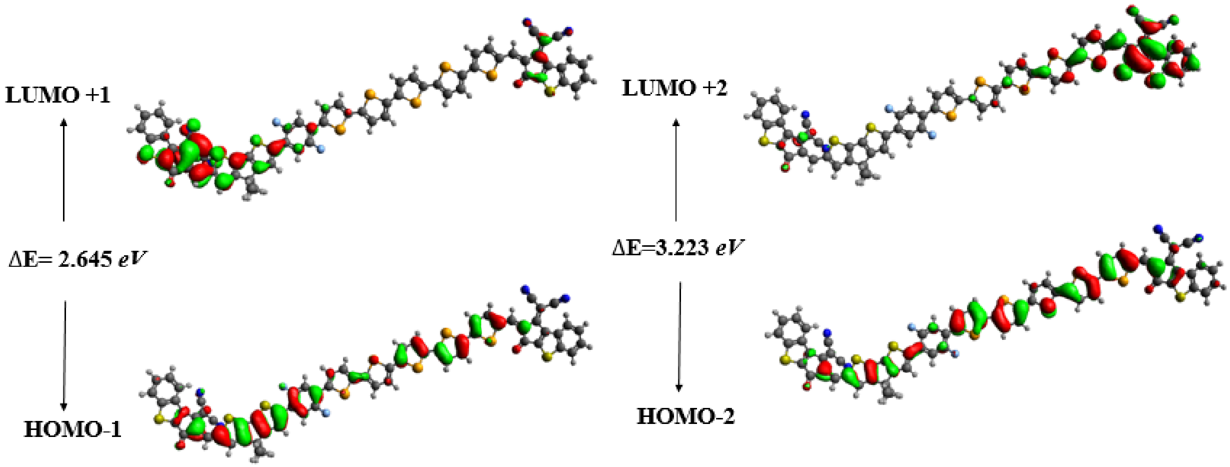

Figure S4: Frontier molecular orbitals of **D5**.

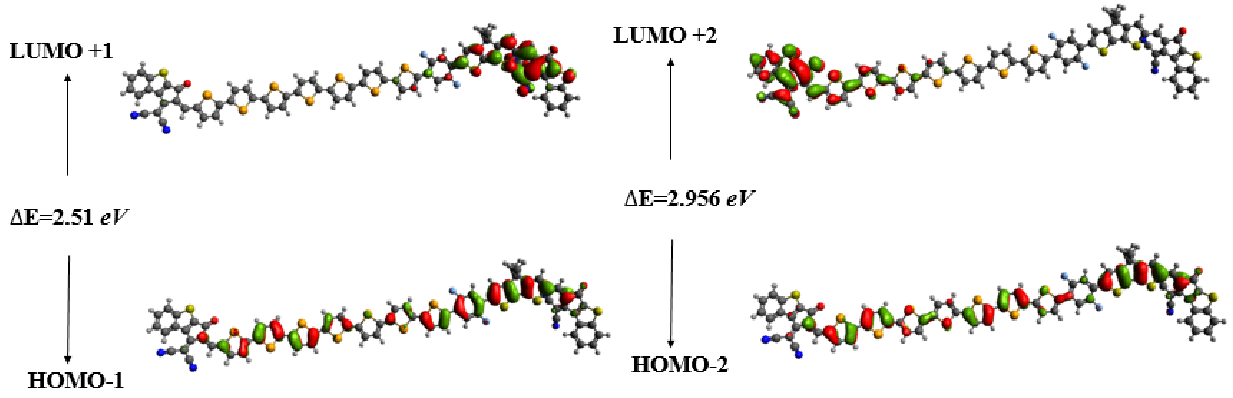

Figure S5: Frontier molecular orbitals of **D7**.

IUPAC Names

D1: 2-((Z)-2-((5-(4-(6-((E)-(1-(dicyanomethylene)-3-oxo-1H-benzo[b]cyclopenta[d]thiophen-2(3H)-ylidene)methyl)-4-isopropyl-4H-cyclopenta[1,2-b:5,4-b']dithiophen-2-yl)-2,5-difluorophenyl)selenophen-2-yl)methylene)-3-oxo-2,3-dihydro-1H-benzo[b]cyclopenta[d]thiophen-1-ylidene)malononitrile

D2: 2-((Z)-2-((5'-(4-(6-((E)-(1-(dicyanomethylene)-3-oxo-1H-benzo[b]cyclopenta[d]thiophen-2(3H)-ylidene)methyl)-4-isopropyl-4H-cyclopenta[1,2-b:5,4-b']dithiophen-2-yl)-2,5-difluorophenyl)-[2,2'-biselenophen]-5-yl)methylene)-3-oxo-2,3-dihydro-1H-benzo[b]cyclopenta[d]thiophen-1-ylidene)malononitrile

D3: 2-((Z)-2-((5''-(4-(6-((E)-(1-(dicyanomethylene)-3-oxo-1H-benzo[b]cyclopenta[d]thiophen-2(3H)-ylidene)methyl)-4-isopropyl-4H-cyclopenta[1,2-b:5,4-b']dithiophen-2-yl)-2,5-difluorophenyl)-[2,2':5',2''-terselenophen]-5-yl)methylene)-3-oxo-2,3-dihydro-1H-benzo[b]cyclopenta[d]thiophen-1-ylidene)malononitrile

D5: 2-((Z)-2-((5''''-(4-(6-((E)-(1-(dicyanomethylene)-3-oxo-1H-benzo[b]cyclopenta[d]thiophen-2(3H)-ylidene)methyl)-4-isopropyl-4H-cyclopenta[1,2-b:5,4-b']dithiophen-2-yl)-2,5-difluorophenyl)-[2,2':5',2'':5'',2''':5''',2''''-quinqueselenophen]-5-yl)methylene)-3-oxo-2,3-dihydro-1H-benzo[b]cyclopenta[d]thiophen-1-ylidene)malononitrile

D7: 2-((Z)-2-((5''''''-(4-(6-((E)-(1-(dicyanomethylene)-3-oxo-1H-benzo[b]cyclopenta[d]thiophen-2(3H)-ylidene)methyl)-4-isopropyl-4H-cyclopenta[1,2-b:5,4-b']dithiophen-2-yl)-2,5-difluorophenyl)-[2,2':5',2'':5'',2''':5''',2'''':5'''',2''''':5''''',2''''''-

sepiselenophen]-5-yl)methylene)-3-oxo-2,3-dihydro-1H-benzo[b]cyclopenta[d]thiophen-1-ylidene)malononitrile

Acceptor-1: 1,4-Difluoro-benzene

Acceptor-2: 2-(2-Methylene-3-oxo-2,3-dihydro-benzo[b]cyclopenta[d]thiophen-1-ylidene)-malononitrile

π-spacer-1: 2-Methyl-selenophene

π-spacer-2: 4-Isopropyl-2-methyl-4H-cyclopenta[2,1-b;3,4-b']dithiophene
